# Clinical cases series and pathogenesis of Lamb-Shaffer syndrome in China

**DOI:** 10.1186/s13023-024-03279-7

**Published:** 2024-07-29

**Authors:** Ruofei Lian, Gongao Wu, Falin Xu, Shichao Zhao, Mengchun Li, Haiyan Wang, Tianming Jia, Yan Dong

**Affiliations:** 1 Department of Pediatrics, The Third Affiliated Hospital of Zhengzhou University, No. 7, Kangfu Front Street, Erqi District, Zhengzhou, Henan Province 450052 China; 2 Henan Key Laboratory of Child Brain Injury and Henan Pediatric Clinical Research Center, the Third Affiliated Hospital and Institute of Neuroscience, Zhengzhou, China

**Keywords:** Lamb-Shaffer syndrome, *SOX5* gene, Global developmental delay, Whole exome sequencing, Neurodevelopmental disorder

## Abstract

**Background:**

Lamb-Shaffer syndrome (LAMSHF, OMIM: 616803) is a rare neurodevelopmental disorder characterized by global developmental delay, intellectual disability, poor expressive speech, which is attributed to haploinsufficiency by heterozygous variants of *SOX5* gene (SRY-Box Transcription Factor 5, HGNC: 11201) on chromosome 12p12. A total of 113 cases have been reported in the world, however, only 3 cases have been reported.in China. Here, we aimed to report novel variants of *SOX5* gene and provide examples for clinical diagnosis by reporting the clinical phenotype of a series of Chinese patients with LAMSHF.

**Methods:**

This study retrospectively collected the information of families of LAMSHF patients in China. Whole Exome Sequencing (WES) were performed to confirm the diagnosis of 4 children with unexplained developmental delay or epilepsy. A minigene splicing assay was used to verify whether the splice variant affected splicing. Meanwhile, a literature review was conducted to analyze the clinical and genetic characteristics of patients with LAMSHF.

**Results:**

Three of the LAMSHF patients had a de novo heterozygous mutation in the *SOX5* gene respectively, c.290delC (p.Pro97fs*30), chr12:23686019_24048958del, c.1772-1C > A, and the remaining one had a mutation inherited from his father, c.1411C > T (p.Arg471*). The main clinical manifestations of these children were presented with global developmental delays, and one of them also had seizures. And the results of the minigene experiment indicated that the splice variant, c.1772-1C > A, transcribed a novel mRNA product which leaded to the formation of a truncated protein.

**Conclusions:**

Through a comprehensive review and analysis of existing literature and this study showed intellectual disability, speech delay and facial dysmorphisms were common clinical manifestation, while the seizures and EEG abnormalities were rare (21/95, 22.16%). Notably, we represent the largest sample size of LAMSHF in Asia that encompasses previously unreported *SOX5* gene mutation, and a minigene testing have been conducted to validate the pathogenicity of the c.1772-1C > A splice variant. The research further expands the phenotype and genotype of LAMSHF while offers novel insights for potential pathogenicity of genes locus.

## Background

Lamb-Shaffer syndrome (LAMSHF, OMIM: 616803) is a neurodevelopmental disorder caused by chromosome 12 deletions ranging from a few kilobases to several trillion bases, including at least a part of the *SOX5* gene, in addition, frame shift variants, splicing variants, nonsense variants and missense variants also cause the disease, which is autosomal dominantly inherited [1]. Clinical features include global developmental delay, intellectual disability, poor expressive speech, and mild dysmorphic facial features, and additional variable skeletal abnormalities may also be present [2]. *SOX5* (SRY-Box Transcription Factor 5, HGNC: 11201) gene is a protein-coding gene that consists of 22 exons, and belongs to the *SOXD* family of transcription factors, together with *SOX6* (SRY-Box Transcription Factor 6) and *SOX13* (SRY-Box Transcription Factor 13). It is the main causative gene among its family members, which plays a role in various developmental pathways such as cartilage formation and neurological development [3]. Since it was first identified and reported by Lamb et al. in 2012 [4], there have been 113 cases reported in all with only 3 cases from China. In our study, 4 LAMSHF children with global developmental delay as the main clinical manifestation which is the largest sample size study in China. Whole Exome Sequencing (WES) results of their families were also analyzed. This study extends the clinical and genetic spectrum associated with LAMSHF and consolidates evidence that *SOX5* gene mutations lead to variable degrees of intellectual disability, language delay, and other clinical features. Meanwhile, the pathogenicity of the de novo splicing site mutation was verified by a minigene splicing assay.

## Methods

### Participant consent and ethical approval

This study recruited 4 patients from 4 nonconsanguineous Han family, who were diagnosed as LAMSHF by clinical examinations, cranial imaging and gene detection, and treated in the outpatient or inpatient department of the Third Affiliated Hospital of Zhengzhou University. Family members provided their written informed consent to participate in this study. The study followed the CARE guidelines and was followed in accordance with the Declaration of Helsinki [5]. Approval was also obtained from the Ethics Committee of The Third Affiliated Hospital of Zhengzhou University (2021–062-01).

### Whole exome sequencing

#### Extraction of genomic DNA and sequence analysis

For extraction of genomic DNA, 2 mL of peripheral blood (with anticoagulant EDTA) was collected from the patients and their parents. A blood genomic DNA extraction kit (Kangwei Century, Shanghai, China) was used according to the manufacturer’s protocol. After quality control and library establishment were complete, DNA fragments in the target region were enriched and a whole exon library was constructed. The probes were captured by xGen Exome Research Panel vl.0 (IDT Company, USA). An Illumina NovaSeq 6000 series sequencer ((Illumina, San Diego, CA, USA) was used for high-throughput sequencing. After quality control was complete, off-machine data were compared with the human reference genome (GRCh37/hg19) using Burrows-Wheeler Aligner software (version 0.59). Genome Analysis ToolKit software (version 4.0.4.0) was used to filter and screen the detected SNPs and indels to obtain high-quality, reliable variants.

#### Mutation confirmed by Sanger sequencing

Primers were designed for candidate mutation sites, and PCR amplification was performed to verify mutations and short fragment deletions or insertion in positive sites. Up- and downstream primer sequences were 5′- TCATAaGATCTCGCTGGAAAGCTATGACAAAC-3′ and 5′- CCAGCGAGATCtTATGAAGAAAGGAGGTTAGGATTCCA-3′, respectively. The PCR program consisted of 35 cycles of denaturation, annealing, and extension. Takara Ex Taq (No. RR001A, Takara Bio, Inc., Otsu, Japan) was used for amplification. The conditions for PCR are presented in Table 1. PCR products were sequenced using ABI 3730XL (ABI, Carlsbad, CA, USA), analyzed with DNASTAR software (DNASTAR, Inc., Madison, USA), and compared with the mRNA template (SOX5: NM_006940.6).

**Table 1:** The system and conditions of PCR

(1) PCR reaction system			
TaKaRa Ex Taq (5U/μl)		0.25 μl	
10 × *Ex Taq* Buffer (Mg^2+^ plus) (20mM)		5 μl	
dNTP Mixture (2.5mM each)		4 μl	
Template		< 500 ng	
Upstream primer		0.2–1.0 μM (final conc.)	
Downstream primer		0.2–1.0 μM (final conc.)	
Sterilized water		up to 50 μl	
(2) PCR conditions			
Steps	Temperature (℃)	Time	Number of cycles
1	98	10S	35
2	98	10S	
	58	30S	
	72	60S	
3	72	10min	

### Minigene splicing assay

We performed a minigene splicing assay on the mutation site of patient 4 (c.1772-1G > A) to verify whether it affects mRNA splicing at the cellular level. The minigene plasmid was designed to insert between exons 10 and 12 of *SOX5*. The primer sequences of amplified gDNA are shown in Table 2. The 5′ end of intron 13 (with the 441-bp sequence) was joined with the 3′ end of intron 13 (with the 698-bp sequence) to form intron 13, and intron14 was also modified with 368-bp sequence of the 5' end and 354-bp sequence of the 3' end retained. The amplified products were cloned into the pMini-CopGFP vector (Hitrobio Biotechnology, Beijing, China), digested with restriction enzymes 5′-BamHI/3′-XhoI using ClonExpress II One Step Cloning Kit (Vazyme, Nanjing, China). The mutant plasmid was generated by site-directed mutagenesis of the wild-type plasmid using SOX5-MUT-F/R primers. The wild-type and variant minigene plasmids, verified by Sanger sequencing, were transiently transfected into human embryonic kidney 293T cells using Lipofectamine 2000 (Invitrogen, Carlsbad, CA, United States). After 48 h, total RNA was extracted from cells using TRIzol reagent (Cowin Biotech, Jiangsu, China). Primers were designed (MiniRT-F and SOX5-RT-R, see Table 2) for reverse transcription-PCR (RT-PCR) followed by Sanger sequencing. Finally, ExPASy Translate (https://web.expasy.org/translate/) was used to translate nucleotide sequences into protein sequences, and to analyze the effect of the mutations on protein sequence.

**Table 2:** Primer sequence information related to Minigene experiment

Subjects	Sequence information
SOX5-AF	5’ - AAGCTTGGTACCGAGCTCGGATCCGAAGTGCTGGAGTCTCAGAGTCAAGAA - 3’
SOX5-AR	5’ - CAAATAGATGGATGAATTCCGCAAATAAAACTCCTTA - 3’
SOX5-BF	5’ - GGAATTCATCCATCTATTTGAATCTCAATTTTCTC - 3’
SOX5-BR	5’ - ACCTGGCTAGGTATTGTCTCAACAAAGTTGTTAG - 3’
SOX5-CF	5’ - ACCTGGCTAGGTATTGTCTCAACAAAGTTGTTAG - 3’
SOX5-CR	5’ - TTAAACGGGCCCTCTAGACTCGAGTCAGTTGGCTTGTCCTGCAATATGGTTTTC - 3’
SOX5-MT-F	5’ - TCATAAGATCTCGCTGGAAAGCTATGACAAAC - 3’
SOX5-MT-R	5’ - CCAGCGAGATCtTATGAAGAAAGGAGGTTAGGATTCCA - 3’

### Literature review

We searched PubMed (https://pubmed.ncbi.nlm.nih.gov/), Online MIM (OMIM; https://omim.org/), Genetic and Rare Diseases Information Center (https://rarediseases.info.nih.gov/), Genetics Home Reference (https://ghr.nlm.nih.gov/), GeneReviews (https://www.ncbi.nlm.nih.gov/books/NBK1116), Chinese Medical Journal Full-text Database (https://www.yiigle.com/index) and Wanfang (Chinese, http://www.wanfangdata.com.cn/) databases using the key words “*SOX5*” or “Lamb-Shaffer syndrome” from January 2012 to December 2023. Case reports of LAMSHF caused by *SOX5* mutations were included. Exclusion criteria were shown as follow: 1) Patients who were not diagnosed as *SOX5* mutation associated LAMSHF; 2) studies published in the format of meetings or as only an abstract. One Hundred Fourteen cases which have been reported up to now were all included in the article.

## Results

### Clinical features

**Fig. 1:**
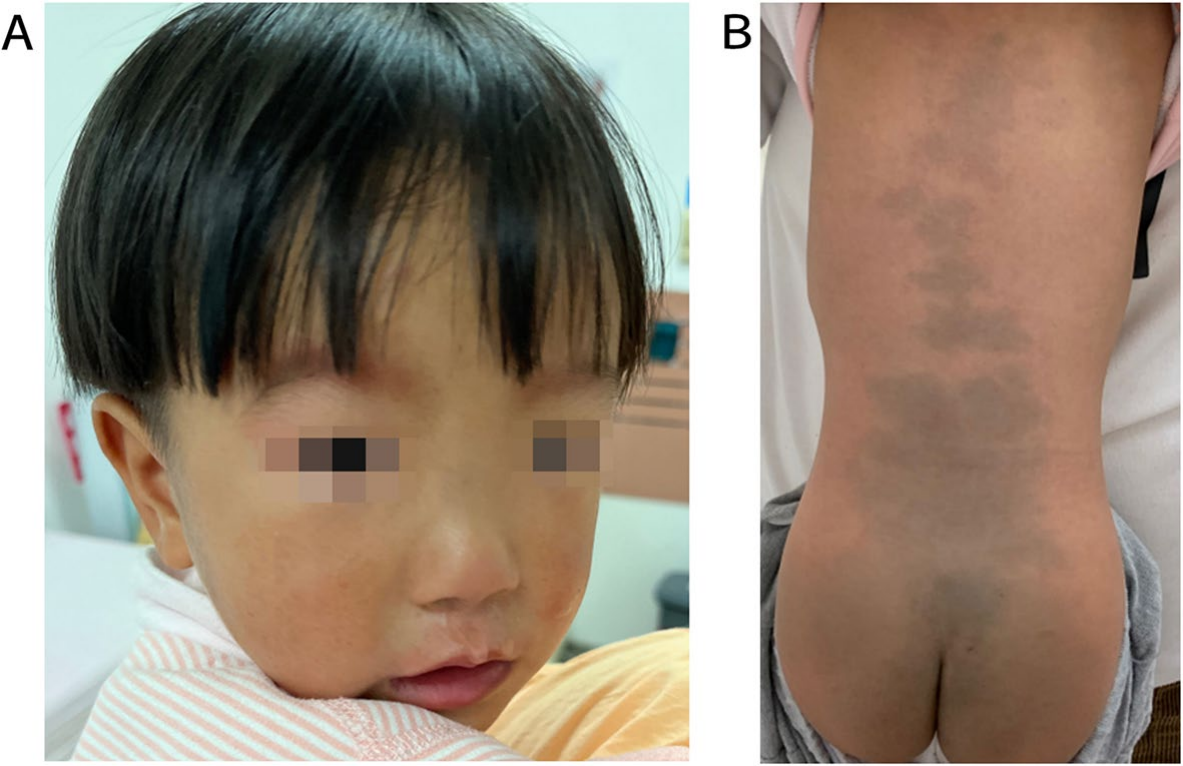
Phenotype of the patient 1 with LAMSHF. **A** Facial features included a low nasal bridge. **B** Large cyanotic birthmarks on the back and buttocks

**Fig. 2:**
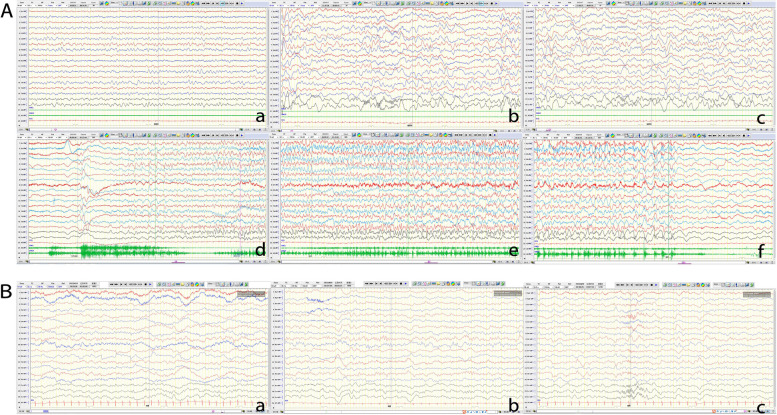
VEEG of the patient 1. **A** VEEG from August 7, 2020, when the patient was aged 3 months and 28days. a. Wakefulness. b,c. Sleep stages. d, c, e. Period of seizure. **B** VEEG from April 19, 2022, when the patient was aged 2 years old. a, b. Wakefulness. c. Sleep stages

Patient 1 was a girl aged 4 years. The child was delivered by cesarean section at term from nonconsanguineous family without apparent personal history and family history. She could roll over at 8 months of age, sit alone at 10 months of age, and stand alone at 18 months of age. At the time of physical examination on admission in April 2022, the child was 2 years old, and she was 80 cm tall (-3SD to -2SD), weighed 9.5 kg (-3SD to -2SD), had a head circumference of 44 cm (-3SD to -2SD). She could sit steadily, stand up with assistance, and walk alone for a few steps, with a low nasal bridge, large cyanotic birthmarks on the back and buttocks, ribs valgus, low muscle strength in all 4 limbs (Fig. 1). In July 2020, at the age of 3 months, the patient had a convulsive seizure uncausedly, accompanied by shaking of the upper limbs and loss of consciousness, lasting 1 to 2 min. The seizures continued at a frequency of 1–2 times per day. Routine blood routine examination and all blood biochemical indices were normal. The patient’s first video electroencephalography (VEEG) at 4 months of age showed low-medium amplitude 2-4Hz slow waves in the occipital region bilaterally during the awake quiet closed-eye state, with 2 tonic clonic seizures monitored. The patient underwent another VEEG at 2 years old, which showed low-medium amplitude 5-6Hz slow waves in the occipital region bilaterally during the awake quiet closed-eye state (Fig. 2). Griffiths Neurodevelopmental Assessment of this patient at 2 years old revealed delayed neurodevelopment equivalent to 1 year old, that she was difficult to perform simple commands, and had poor language comprehension and expression, fine motor skills, eye-hand coordination. The patient had been administered valproic acid, levetiracetam, lamotrigine, clonazepam, and topiramate in turn since seizure episodes, but she continued to experience several dozens per day, and had developed atonic seizures and atypical absence seizures. Rehabilitation and functional training included low frequency impulse current therapy, transcranial magnetic therapy, muscle strength training, and language training intermittently. The patient progressed slowly in development and still lagged behind (Table 3).

**Table 3:** The clinical data of patients diagnosed with LAMSHF in this study

Patient	Gender	Age	Variant	Inherited /de novo	Past medical history	Personal history	Family history	Physical examination	Motor development and cognitive competence	Language and speech	Behavior disorder	Seizures and EEG abnormalities
Patient1	Famale	4 years old	c.290delC (p.Pro97fs*30)	de novo	No significant findings	No significant findings	No significant findings	Short in stature	Developmental delay	Short sentences can be employed	No significant findings	Frequent seizures of multiple types with poor effectiveness of antiseizure medications
Patient2	Male	5 years and 7 months old	chr12:23686019_24048958del	de novo	No significant findings	Little fetal movement and fetal malposition	No significant findings	Short in stature, feet valgus deformity and abnormal of oropharyngeal muscles and salivation	Developmental delay	Poor understanding of language, couldn't say complete sentences	Lack of patience and difficulty in concentrating	No significant findings
Patient3	Male	4 years and 5 months old	c.1411C > T (p.Arg471*)	Inherited	No significant findings	Low-implantation pregnancy, underwent traditional Chinese medicine treatment	Child's father was a severe case of autism	Short in stature	Developmental delay	Regression in language development	No significant findings	No significant findings
Patient4	Famale	3 years and 2 months old	c.1772-1C > A	de novo	No significant findings	No significant findings	No significant findings	Short in stature	Developmental delay	Use simple words consciously, but could not express simple needs with words	No significant findings	No significant findings

Patient 2 was a 5 years and 7 months old boy. There was little fetal movement during his mother’s perinatal period, and he was delivered at term by cesarean section because of fetal malposition. He had feeding difficulties and was 1–2 months delayed compared with normal children in all developmental milestones during growth. At 17 months of age, he was treated for his inability to walk unaided. The patient had no family history and his parents were nonconsanguineous. Physical examination data at the age of 4 years showed that the patient was 100 cm tall (-2SD to -1SD), weighed 13.5 kg (-2SD to -1SD), with his feet valgus deformity, abnormal of oropharyngeal muscles and salivation. No other special features were seen. The child had less active language, and could imitates speech sounds (nonspecific "dada" and "mama"). Because of this, he often used gestures to express his willingness, and had poor understanding of language. The patient could not count numbers. The Griffiths Neurodevelopmental Assessment conducted at the age of 4 revealed global developmental delay. In addition, the parents described his lack of patience and difficulty in concentrating. Results of blood routine examination, all blood biochemical indices, and auxiliary examination were normal. He could walk independently at the age of 20 months, walk unassisted up and down stairs at the age of 3 years, and jump with two feet off the floor at the age of 4 years after musccould strength training and core training, lip muscle training, language training. The boy could use of "dada", "mama", plus 1–4 other words, and express simple demands by body language at follow-up visit in May 2024 (Table 3).

Patient 3 was a boy at the age of 4 years and 5 months. Because of the diagnosis of low-implantation pregnancy, his mother was treated with traditional Chinese medicine to protect the fetus from the third month of pregnancy until he was delivered at term. The boy had global developmental delay after birth, as evidenced by that he could stand up and turn over at the age of 5 months, sit at the age of 8 months, crawl at the age of 16 months, stand and walk without assistance at the age of 2 years and 4 months. The child had a regression in language development. He could imitates speech sounds "dada" and "mama" at 2 years and 5 months, but was lost after 2 months, only left "babbling" sounds. He was not diagnosed with autism due to the absence of other classic symptoms, such as stereotyped behaviors and preoccupation or fascination with a singel object or subject, and his scores of Child Autism Rating Scale (CARS) and the Autism Diagnostic Observation Schedule (ADOS) were normal, although his language function declined and his father had severe autism. His mother and sister are healthy, and his parents were nonconsanguineous. Physical examination showed the patient was 91 cm tall (-2SD to -1SD), weighed 13.5 kg (-1SD to 0) at 2 years and 10 months old. His squint had not received treatment. The motor and language development of the patient improved gradually after received rehabilitation therapy, such as muscle strength training, and language training (Table 3).

Patient 4 was a 3 years and 2 months old girl with global developmental delay and language loss. She was a term infant delivered by cesarean section with normal fetal period, and her parents were nonconsanguineous. At the time of physical examination on admission, she was 1 year and 2 months old without specific facial features. The result showed that she was 73.3 cm tall (-2SD to -1SD), and weighed 8.4 kg (-2SD to -1SD). According to the Griffiths Neurodevelopmental Assessment, she displayed developmental delay in social interaction, language, eye-hand coordination at 2 years and 1 month, especially language. The child could use 20–30 words, but could not listen stories attentionally, use words express simple demands, and play games with peers. Additionally, the child could not complete assigned tasks accurately. The child showd no significant progress after language training and expressed herself rarely (Table 3).

### Whole exome sequencing

The WES test results showed that the *SOX5* gene (NM_152989) of all 4 patients had novel mutations. Three patients had a de novo heterozygous mutation: patient 1, c.290delC (p.Pro97fs*30), patient 2, chr12:23686019_24048958del and patient 4, c.1772-1C > A, and patient 3 had a mutation inherited from his father c.1411C > T (p.Arg471*).

In patient 1, WES revealed a novel heterozygous c.290delC (p.Pro97fs*30) variant in the *SOX5* gene. Pathogenicity analysis performed according to American College of Medical Genetics and Genomics 2019 guidelines (ACMG) [6] indicated that the novel variant was pathogenic (PVS1 + PS2_Moderate + PM2_Supporting). This is the first case of LAMSHF with this variant locus, which is not reported in the Single Nucleotide Polymorphism Database (dbSNP, https://www.ncbi.nlm.nih.gov/SNP/), 1000 Genomes Project (1000G, https://www.internationalgenome.org/), or Genome Aggregation Database (gnomAD, http://www.gnomad-sg.org/) (PM2_Supporting). Because of this mutation, from the 97th amino acid of the encoded protein, the translation of the protein was terminated after another 30 amino acids. These 30 amino acids were frameshift, and the sequence was different from the original, which may affect the function of the subsequent high-mobility-group (HMG) box domain (PVS1), Moreover, it is a de novo variant, which was absent in other family members (PS2_Moderate).

In patient 2, WES showed a 0.36Mb deletion at 12p12.1, covering exons 2–15 of the *SOX5* gene. The CNV was rated as pathogenic according to ACMG guidelines [6] and involved a RefSeq protein-coding gene. LAMSHF was found in patients with *SOX5* gene haploinsufficiency after searching the ClinGen database. The deletion of exons 2–15 of the *SOX5* gene caused by this mutation seriously affected the expression of the *SOX5* gene, thus causing the disease.

In patient 3, WES revealed an inherited heterozygous c.1411C > T (p.Arg471*) mutation in the *SOX5* gene from his father. This mutation was a nonsense mutation, which was predicted to cause the Arg at position 471 of the encoded protein to be replaced by a stop codon and lead to the premature termination of protein translation, resulting in the truncation of the encoded protein and loss of its normal function (PVS1). To the best of our knowledge, no literature report was found in the Human Gene Mutation Database (HGMD, https://www.hgmd.cf.ac.uk/ac/index.php), and dbSNP, 1000G (PM2_Supporting). And pathogenicity analysis performed according to ACMG [6] indicated that the variant was likely pathogenic (PVS1 + PM2_Supporting).

In patient 4, WES suggested the presence of a novel splice variant in intron 13 (c.1772-1C > A) of the *SOX5* gene that was not present in other family members (PS2_Moderate). This variant was a classical splice site variant and the associated disease was loss-of-function, which was located in the biologically significant transcript and was not located in the last coding exon or the last 50bp of the penultimate coding exon. It was predicted to activate nonsense mediated mRNA degradation, thereby affecting the function of the protein encoded by the gene (PVS1). And no data about this mutation were found in the gnomAD and 1000G (PM2_Supporting). Pathogenicity analysis performed according to ACMG [6] indicated that the novel variant was pathogenic (PVS1 + PS2_Moderate + PM2_Supporting).

### Minigene testing

**Fig. 3:**
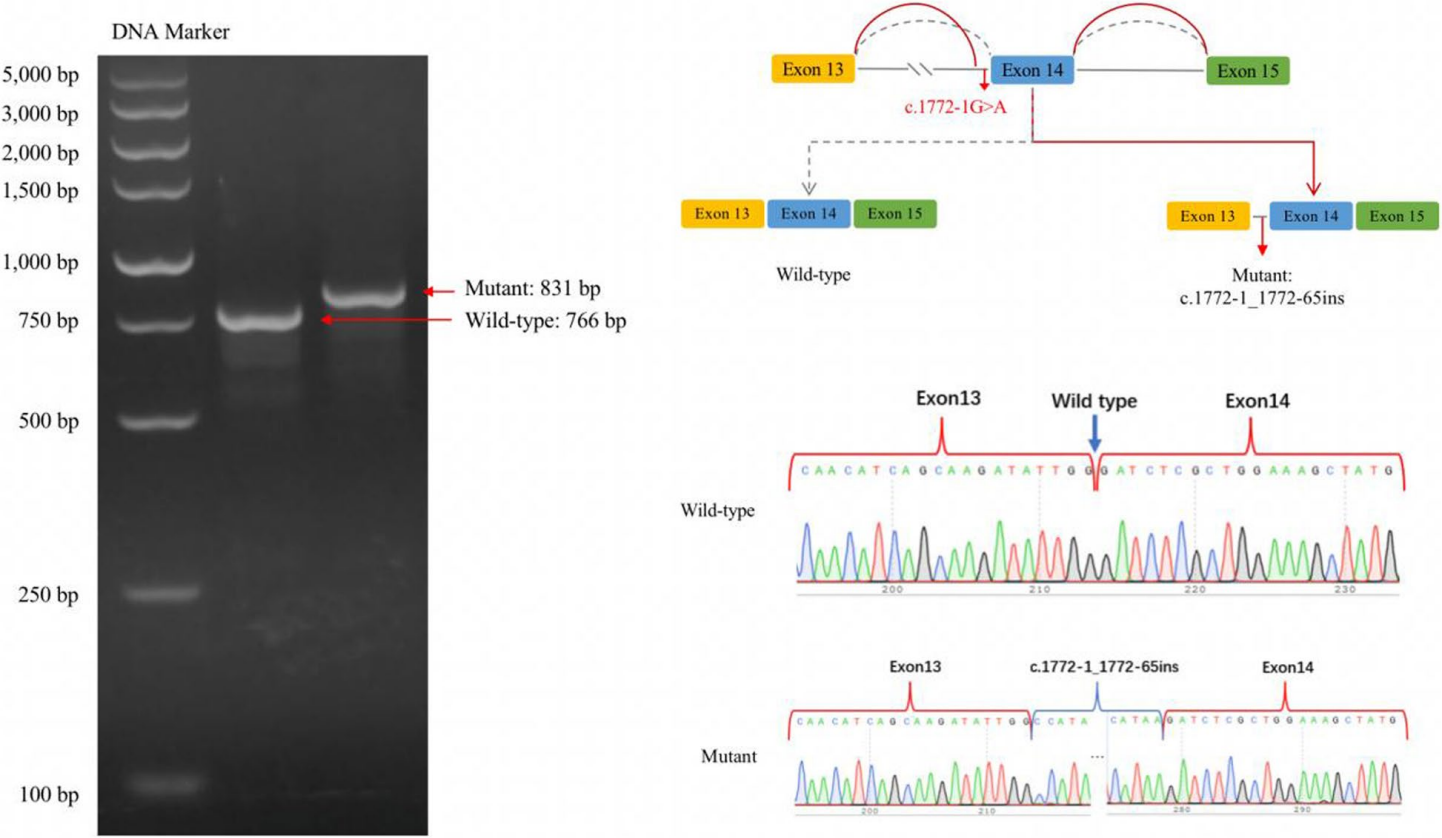
Splicing analysis using a minigene splicing assay. The variant plasmid transcribed a novel mRNA product that lead to the retention of a partial sequence (65 bp) in intron13. The mRNA was expressed as NM_006940.6: c.1772-1_1772-65ins (p.Gly591AlafsTer22)

The transcribed mRNA sequence from the wild-type plasmid contained complete exon 13–15. The variant plasmid transcribed only one mRNA product, with a 65 bp retained sequence in intron13. The product resulted in a frameshift variant and premature termination codon, which may lead to an amino acid shift to form a truncated protein. And the mRNA was expressed as NM_006940.6: c.1772-1_1772-65ins (p.Gly591AlafsTer22) (Fig. 3).

### Pooled analysis of the literature

**Fig. 4:**
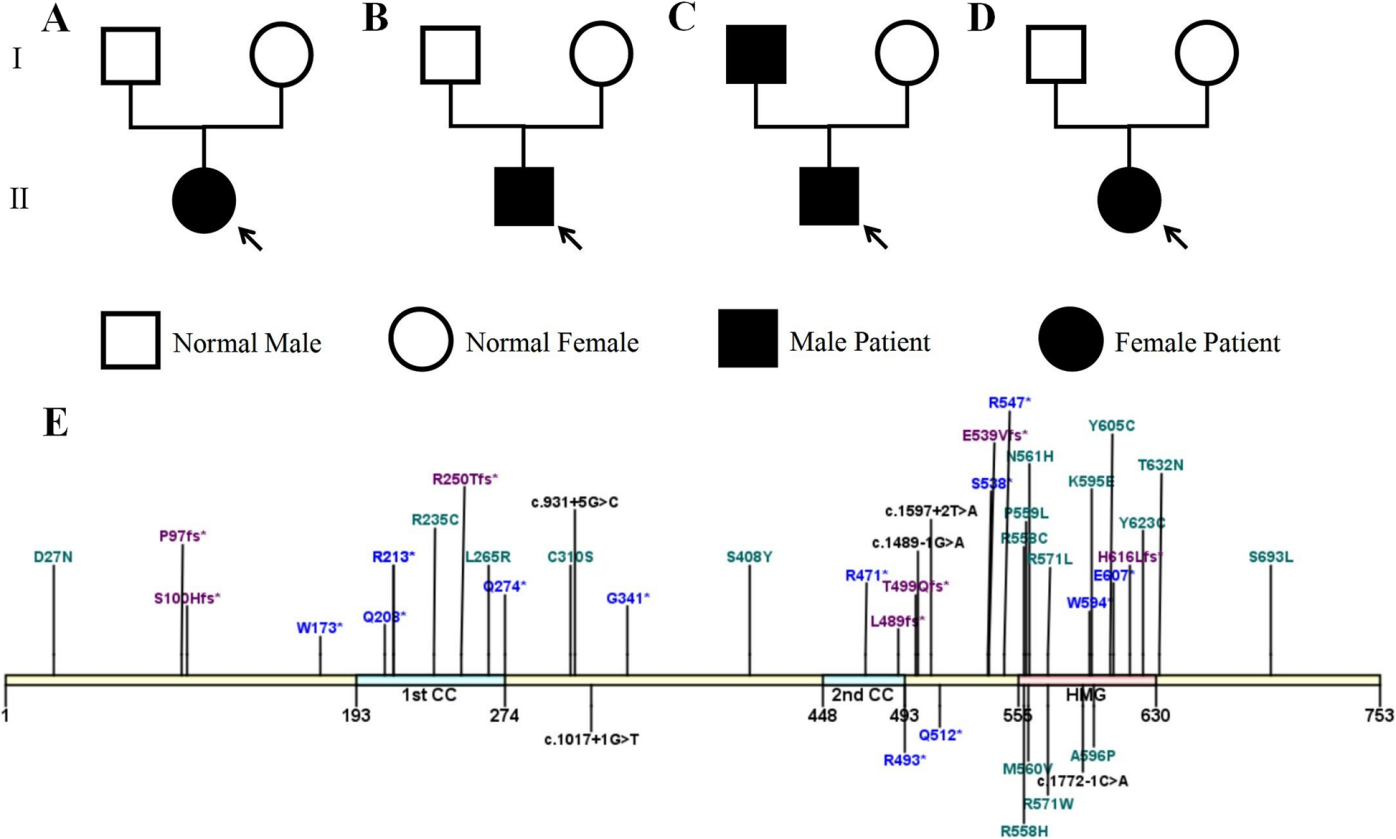
Genealogy map of the 4 patients’s family (**A** Patient 1, **B** Patient 2, **C** Patient 3, **D** Patient 4). Structure of SOX5 protein (**E**, searched onUniPort, https://www.uniprot.org/). Location of point variants reported here and previously on the longest SOX5 isoform. Protein and domain residueboundaries are indicated underneath the schematic (CC, coiled-coil domain, HMG, high-mobility-group box domain). Blue, nonsense variants. Purple, frameshift variants. Green, missense variants. Black, splicing variant

We searched 114 patients with LAMSHF from the literature during January 2012 to February 2024, and added 4 patients in this paper are summarized together in Table 4 [1–4, 7–18]. Almost all of the reported cases had developmental delay or intellectual disability (117/118, 99.15%). Other common clinical manifestations included speech delay (77/95, 81.05%), facial dysmorphisms (67/98, 68.37%), and behavioral problems (67/98, 68.37%) including autism, attention deficit and hyperactivity disorder (ADHD), disruptive and obsessive–compulsive behaviors, and anxiety and insomnia. Ocular signs (72/114, 63.16%) such as myopia, strabismus, amblyopia, bilateral optic nerve hypoplasia and optic nerve atrophy were usually described. Hypotonia (61/112, 54.46%), structural brain abnormalities (25/70, 35.71%), and skeletal deformities (33/100, 33.00%) such as scoliosis, vertebral fusion and dwarfism were common, while the seizures and EEG abnormalities were rare (21/95, 22.16%). Combining the genotypes of those 118 patients, a total of 50 cases were reported to have chromosome fragment deletion with the number of deletions ranging from 9b to 12.09Mb, but all of them involved *SOX5* transcription factor [3, 4, 7, 11, 14, 17, 18]. Since Nesbitt et al. irst reported the mutation within *SOX5* transcription factor in 2015, a total of 43 cases of such gene variation have been reported (Fig. 4), including 18 missense variants, 13 nonsense variants, 7 frame shift mutations, and 5 splice variants, and all of these mutations were caused by single base changes [1, 2, 8, 10, 13, 15–17]. In addition to this, there was also one patient with chromosomal translocation [9].

**Table 4:** Summary of phenotypes in families with *SOX5* mutations

Reference	Variant	Inherited/ de novo	ID/DD	Speech delay	Facial dysmorphisms	Behavioral problems	Ocular signs	Hypotonia	Skeletal deformities	Structural brain abnormalities	Seizures and EEG abnormalities	Other system anomalies
			117/118 99.15	77/95 81.05	67/98 68.37	67/100 67.00	72/114 63.16	61/112 54.46	33/100 33.00	25/70 35.71	21/95 22.16	37/107 34.56
Lamb et al. (2012) [4]	8 subjects with heterozygous deletions that only involved *SOX5* and ranged in size from 72 to 466 kb. 7 subjects with 12p deletions encompassing multiple genes including *SOX5*, ranging from 1.4 Mb to 12.1 Mb and including 8 to 63 genes. 1 subject with no other clinically significant gains or losses of DNA.	3/9 6/9	16/16	13/14	12/16	10/14	10/16	8/15	4/7	6/10	3/16	2/9
Schanze et al. (2013) [3]	3 subjects identified deletions have different breakpoints and range in size from 120 kb to 4.9 Mb, the smallest deletion helps to narrow down the critical region to a genomic segment encompassing only one gene, *SOX5*.	0/2 2/2	3/3	3/3	2/3	1/3	2/3	1/3	2/3	1/2	0/3	2/3
Lee et al. (2013) [11]	1 subject with de novo 53 kb interstitial deletion at 12p12.1. 1 subject with 3.2 Mb deletion at 12p12.2 p12.1.	0/1 1/1	2/2	2/2	2/2	0/2	1/2	2/2	1/2	1/2	–	1/2
Quintela et al. (2015) [7]	1 subject with inherited 16p13.11-p12.3 duplication and a de novo 12p12.1 deletion affecting *SOX5*.	1/1 0/1	1/1	1/1	1/1	1/1	1/1	0/1	0/1	0/1	0/1	0/1
Nesbitt et al. (2015) [2]	c.1021G > T, p.(Gly341*)	0/1 1/1	1/1	1/1	1/1	1/1	1/1	0/1	1/1	0/1	0/1	0/1
Fukushi et al. (2018) [9]	t (12;20) (p12.1; p12.3)	0/1 1/1	1/1	1/1	1/1	1/1	0/1	0/1	1/1	0/1	0/1	0/1
Gkirgkinoudis et al. (2020) [8]	c.1783A > G, p.(Lys595Glu)	0/1 1/1	0/1	0/1	0/1	0/1	0/1	0/1	1/1	0/1	0/1	0/1
Zawerton et al. (2020) [1]	8 subjects with microdeletions ranged from 43.7 kb to 1.7 Mb and involved different breakpoints. 33 subjects with 23 distinct point variants: c.518G > A, p.(Trp173*) c.622C > T, p.(Gln208*) c.637C > T, p.(Arg213*) c.747_748del,p.(Arg250Thrfs*36) c.820C > T, p.(Gln274*) c.1465dup, p.(Leu489Profs*3) c.1477C > T, p.(Arg493*) c.1489-1G > A c.1597 + 2T > A c.1613C > G, p.(Ser538*) c.1678A > G, p.(Met560Val) c.1681A > C, p.(Asn561His) c.1711C > T, p.(Arg571Trp) c.1712G > T, p.(Arg571Leu) c.1782G > A, p.(Trp594*) c.1786G > C, p.(Ala596Pro) c.1814A > G, p.(Tyr605Cys) c.1819G > T, p.(Glu607*) c.1868A > G, p.(Tyr623Cys) c.703C > T, p.(Arg235Cys) c.928T > A, p.(Cys310Ser) c.1895C > A, p.(Thr632Asn) c.2078C > T, p.(Ser693Leu)	9/37 28/37	41/41	21/26	31/37	17/25	19/37	22/37	9/37	–	8/36	5/37
Cao et al. (2021) [13]	c.1495delA, p.(Thr499Glnfs*5)	0/1 1/1	1/1	1/1	1/1	0/1	0/1	0/1	1/1	0/1	1/1	0/1
Innella et al. (2021) [10]	5 subjects with *SOX5* deletion, these deletions range in size from around 50–300 kb and involve different breakpoints. 1 subject with variant of c.1672C > T, p.(Arg558Cys).	3/5 2/5	6/6	–	4/6	4/6	4/6	1/6	3/6	2/6	4/6	2/6
Zhang et al. (2022) [12]	Seq[GRCh37]del(12)(p12.1;p11.1)	0/1 1/1	1/1	1/1	1/1	0/1	0/1	0/1	1/1	0/1	0/1	0/1
Zhu et al. (2023) [16]	c.1477C > T, p.(R493*)	0/1 1/1	1/1	1/1	1/1	0/1	0/1	0/1	1/1	0/1	0/1	1/1
Mahmud et al. (2023) [14]	271kb de novo deletion in 12p12.1	0/0 0/0	1/1	1/1	0/1	0/1	1/1	1/1	1/1	0/1	0/1	1/1
Edgerley et al. (2023) [17]	99 kb Del ex7–9 (12:23576437–23,675,507), 53 kb Del ex4–8 (12:23765508–23,919,836), 103 kb Del ex 4–6 (12:23718514–23,841,144), 425 kb Del 1–3 (12:23,924,962–24,349,949), 556 kb Del ex13–14 (12:23944863–24,501,142), 491 kb Del ex1–6 12p12.1 (12:24,097,797–24,654,076), c.1616_1617del,p.(Glu539ValfsTer5) c.637C > T,p.(Arg213*) c.1534C > T,p.(GLn512*) c.1477C > T,p.(Arg493*) c.1676C > T,p.(Pro559Leu) c.1786G > C,p.(Ala596Pro)	3/14 11/14	16/16	16/16	–	11/16	13/16	8/15	3/11	5/16	–	8/16
Tenorio-Castano et al. (2023) [19]	c.79G > A, p.(Asp27Asn) c.298del, p.(Ser100HisfsTer40) c.794T > G, p.(Leu265Arg) c.1223C > G, p.(Ser408Ter) c.1535del, p.(Gln512ArgfsTer11) c.1673G > A, p.(Arg558His) c.1712G > A, p.(Arg571Gln) c.1847_1851delACCTGinsT, p.(His616LeufsTer99) c.931 + 5G > C c.1017 + 1G > T chr12:21000550–27266871 6.27 Mb Del chr12:22132484–24812938 2.68 Mb Del chr12 23356757 23815173 458 kb Del chr12 23413378 25448174 2.03 Mb Del chr12:24076457–24358695 282 kb Del	0/20 20/20	20/20	9/20	7/20	20/20	19/20	18/20	3/20	10/20	4/20	15/20
Wang et al. (2023) [20]	c.1639C > T, p.(Arg547*)	0/1 1/1	1/1	1/1	0/1	0/1	0/1	0/1	1/1	0/1	0/1	0/1
Sajewicz-Radtke U et al. (2024) [18]	a microdeletion at chromosome 12p12.3p12.1	0/1	1/1	1/1	1/1	0/1	0/1	0/1	0/1	0/1	0/1	0/1
This study	c.290delC, p.(Pro97fs*30) chr12:23686019_24048958del c.1411C > T, p.(Arg471*) c.1772-1C > A	1/4 3/4	4/4	4/4	2/4	1/4	1/4	0/4	0/4	0/4	1/4	0/4

The symbol “–” indicates not mentioned

*Abbreviations*: *DD* developmental delay, *ID* intellectual disability

## Discussion

Lamb-Shaffer syndrome is caused by a partial or complete deletion of the *SOX5* gene on chromosome 12p12 resulting in haploinsufficiency, and the occurrence of frame shift variants, splicing variants, nonsense variants and missense variants can also affect its haploinsufficiency effect and cause the disease [1, 4]. In 2012, by the use of oligonucleotide-based array comparative genomic hybridization (array CGH) to test 24,081 probands referred for developmental delay or intellectual disability, Lamb et al. identified 10 patients with large deletions of chromosome 12p12 that involved all or part of the *SOX5* gene and additional genes [4]. And the reported patients with haploinsufficiency of the *SOX5* gene had common features included developmental delay, intellectual disability, speech delay and dysmorphisms such as strabismus, frontal bossing, ear abnormalities, and low nasal bridge. This disease is extremely rare, that a total of 114 patients have been reported, including only 3 Chinese patients from 2012 to 2024. In this study, we present a comprehensive analysis of LAMSHF in Asia, comprising the largest sample size to date. Our findings include four distinct types of genetic variants encompassing chromosomal segment deletions, truncating mutations, splice mutations, and missense mutations, among the cohort of patients, patient 3 exhibited a variant locus inherited paternally, yet presented with a distinct clinical phenotype. This study contributes to the existing phenotype database for LAMSHF and provides valuable evidence for further investigations into gene-phenotype correlations. We conducted a comprehensive analysis of the clinical presentations and genotypes of all reported patients, revealing evidence of incomplete penetrance in certain features. This suggests that the manifestation of *SOX5* haploinsufficiency may vary among individuals with different genetic backgrounds or that specific variants retain partial activity. However, our investigation into the correlation between variant types and clinical features did not yield a definitive genetic-phenotypic association.

*SOX* transcription factors play many different roles during development, including regulating the state of stem cells, directing their differentiation, and influencing local chromatin expression. The family of *SOX* transcription factors has an HMG structural domain that mediates DNA binding and bending, nuclear transport, and protein–protein interactions. In the *SOX* subfamily, other than the HMG, the other structural domains of proteins are similar but not identical, and the *SOXD* factor contains a coiled helix structural domain that mediates its homodimerization or heterodimerization with other *SOXD* factors and stabilizes adjacent HMG binding sites on DNA [21]. Among the 20 *SOX* transcription factors in vertebrates, members of the *SOXC*, *SOXG*, *SOXD* and *SOXE* families play key roles in the development of the neural crest and formation of neural crest-derived structures, including the craniofacial complex formation [22].

*SOX5* produces several tissue-specific alternate transcripts by priming different promoter sites or alternative splicing: the longest form, encoding a 763 amino acid protein, initially called *L-SOX5* (NM_006940) and later more commonly referred to as *SOX5*, is the major form in brain that plays an important role in the formation and differentiation of the human fetal brain [1, 2]. Studies have showed that *SOX5* is required for newborn deep-layer neurons to migrate and laminar-specific neuronal identities to be refined during postmigratory differentiation [21]. Both *SOX5* and *SOX13* are expressed during neural crest formation, but only the role of *SOX5* has been reported. Loss of function experiments demonstrated that *SOX5* is necessary for neural crest, placode, and neural plate border formation, suggesting that maintaining the correct level of *SOX5* expression is key to proper neural crest formation [22]. A recent study showed that *SOX5* and *SOX6*, which is fellow genus *SOXD* transcription factor family with *SOX5*, controls and maintains neurogenic ecological niche of the subgranular layer of hippocampus dentate gyrus by activating neural stem cells (NSCs) in a state of reversible quiescence, and the deletion of *SOX5* affects NSCs response to environmental enrichment, affecting the neurogenesis process [23]. The shortest form, *S-SOX5* (NM_178010) encodes a protein corresponding to the C-terminal half of *L-SOX5* and lacks the convoluted helix domain at the N-terminal end of *L-SOX5*. Genome Wide Association Study studies suggest that the *SOX5* locus is associated with male infertility [24], and the high expression level of *S-SOX5* in the testis implies that it plays an important role in regulating expression of the genes that are essential for sperm function and male fertility [25, 26]. *SOX5* also works in concert with transcription factors such as *SOX6* (*SOXD* group), *SOX9* (*SOXE* group) and they are called the chondrogenic trio because of their necessity in chondrocyte growth and differentiation, and gain-of-function studies have demonstrated that the trio is sufficient to convert progenitor cells into chondrocytes [27]. These genes are continuously active in the chondrocyte lineage from the pre-mesenchymal phase of the growth plate to the pre-hypertrophic phase [28].

Among the four patients we reported in this article, one patient had epilepsy as the prominent manifestation, and the other three patients had delayed and regressive language development. In previous studies of LAMSHF, the prevalence of phenotypes with epilepsy or abnormal EEG was found to be the lowest at 22.16%, and no comprehensive description of their EEG characteristics was provided. Additionally, the involvement of the *SOX5* gene in epilepsy pathogenesis was not addressed. In this study, we present a case of early-onset epilepsy in an infant at 3 months of age. The electroencephalogram (EEG) revealed bilateral occipital low-amplitude slow waves, and seizure type progression included tonic–clonic seizures evolving into atonic seizures and atypical absence seizures. Despite administration of various antiseizure medications, seizure control was not satisfactory. Diego A Forero carried out meta-analyses and analyses of convergence for available genome-wide expression studies (GWES) for epileptogenesis in humans and in mouse, rat, zebrafish and fruit fly models. He found that a large number of top genes for epileptogenesis are regulated by key transcription factors, such as *MEF2* (myocyte enhancer factor 2) and *SOX5*, which are interesting candidates for the pathogenesis of epilepsies [29]. Tumienė and Lee et al. had also reported *SOX5* gene variants in the statistics of genetic study of epilepsy [30, 31]. According to these studys, we speculate that *SOX5*, as a transcription factor gene, may be involved in the regulation of the expression of known epilepsy genes, and this interesting gene deserves further investigation. It has been reported that patients with HMG domain missense variants tended to have milder language deficits, but this finding requires confirmation with larger patient cohorts [1].

In addition, the types of gene mutations in the four patients were frameshift mutation, chromosome deletion mutation, mistranslation mutation, and splicing mutation and patient 3 inherited it from his father. Minigene assay was used to confirm the pathogenicity of the splice site (c.1772-1C > A), and the results showed that the variant transcribed a new mRNA product, which resulted in partial retention (65 bp) of intron13 in the mature mRNA. By reviewing the 114 cases reported in the previous literature, although we did not reveal a clear genotype–phenotype correlation, we found that there was considerable variation in clinical features and severity among different individuals. Patients with de novo mutations have more severe clinical symptoms, earlier age of onsets, higher frequency of multi-organ involvements, and poorer prognoses than those with familial inheritance. It does seem to be somewhat evident that truncating sequence variants are more likely to result in smaller growth parameters than intragenic deletions, which was also validated in the four patients we reported. And the point mutation (or balanced translocation) patients are the ones who are more likely to be small with small heads (but not exclusively so) [17]. Zawerton et al. suggest that brain growth is frequently altered in the patient cohort with sequence variation [1]. Previous studies have shown that the main challenge around learning (and in managing behavior) for patients with LAMSHF is usually around speech and communication which clearly compounds any underlying learning difficulty [17]. However, the relationship between the mutation types and phenotypes still needs further multi-center and large sample studies.

Currently, there was no effective treatment available for LAMHSF, and children diagnosed with this disease could receive symptomatic treatments. For instance, antiseizure medications might be administered to patients experiencing seizure attacks based on their clinical manifestations, with adjustments according to the treatment response. Rehabilitation training could be provided to address motor delay in affected individuals. However, it should be noted that the antiseizure medications were found to be ineffective in treating the LAMHSF children reported in this study. Following rehabilitation treatment, significant improvements were observed in gross motor skills and fine motor development of these children. However, language delay remaind a challenging issue as they primarily rely on body language and eye contact to express emotions and needs. In 2024, Sajewicz-Radtke et al. [18] reported the establishment of a comprehensive multidisciplinary care model for a 7-year-old patient with LAMHSF which emphasized integrating language therapy into the overall treatment process. This suggested that adopting a holistic approach involving geneticists, psychologists, speech therapists, physiotherapists, optometrists as well as parents was crucial for providing optimal developmental opportunities for children with rare genetic diseases. It was anticipated that future efforts will lead to the establishment of an even more comprehensive and sustainable treatment model. Genetic testing is recommended for children presenting unclear developmental delays along with facial deformities or behavioral problems in order to confirm diagnosis. Families affected by LAMHSF should consider prenatal diagnosis.

## Conclusions

In this study, we diagnosed 4 LAMSHF patients by WES technology, and none of the pathogenic sites (c.290delC (p.Pro97fs*30), c.1411C > T (p.Arg471*), c.1772-1C > A and chr12:23686019_24048958del) have been reported previously. The summary and analysis of available reports showed intellectual disability (117/118, 99.15%), speech delay (77/95, 81.05%) and facial dysmorphisms (67/98, 68.37%) were common clinical manifestations in the reported cases, while the seizures and EEG abnormalities were rare (21/95, 22.16%). This study is the largest sample size of LAMSHF in Asia currently, including one child with epilepsy which is infrequent in this disease, and another child exhibiting an inherited variant but presenting with a distinct phenotype with his father. Additionally, we conducted a minigene testing to validate the pathogenicity of the c.1772-1C > A splice site variant. The research further expands the phenotype and genotype of LAMSHF while offers novel insights for potential pathogenicity of genes locus.

## Data Availability

The dataset supporting the conclusions of this article is available in: https://www.ncbi.nlm.nih.gov/clinvar/, accession numbers: SCV004030253. According to national legislation/guidelines, specifically the Administrative Regulations of the People's Republic of China on Human Genetic Resources (https://www.gov.cn/zhengce/content/2019-06/10/content_5398829.htm, http://english.www.gov.cn/policies/latest_releases/2019/06/10/content_281476708945462.htm), no further datasets presented in this article are readily available. Requests to access the datasets should be directed to the corresponding author.
